# Cleaning and dressing the eye after surgery

**Published:** 2016

**Authors:** Sue Stevens

**Affiliations:** Former Nurse Advisor, Community Eye Health Journal, International Centre for Eye Health, London School of Hygiene and Tropical Medicine, London, UK.

**Figure:**
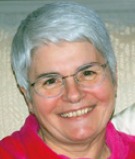
Sue Stevens

## 1 Cleaning the eyelids

### 

#### Before you start

- Wash your hands (and afterwards too).
- Wear gloves if available/required.
- Position the patient comfortably with head supported.
- Avoid distraction for yourself and the patient.
- Ensure good lighting.
- Explain to the patient what you are going to do.

#### You will need

- Sterile gauze swabs.
- A pre-made salt solution suitable for eyes, if available. You can make up your own: dissolve 1 heaped teaspoonful of salt or sodium bicarbonate in a jug containing 500 ml of boiled water (half a litre) and allow the solution to cool.
- Pour a very small amount of the solution into a small sterile pot on a clean surface.

### Method

#### 1 The eyelashes

**Figure 1:**
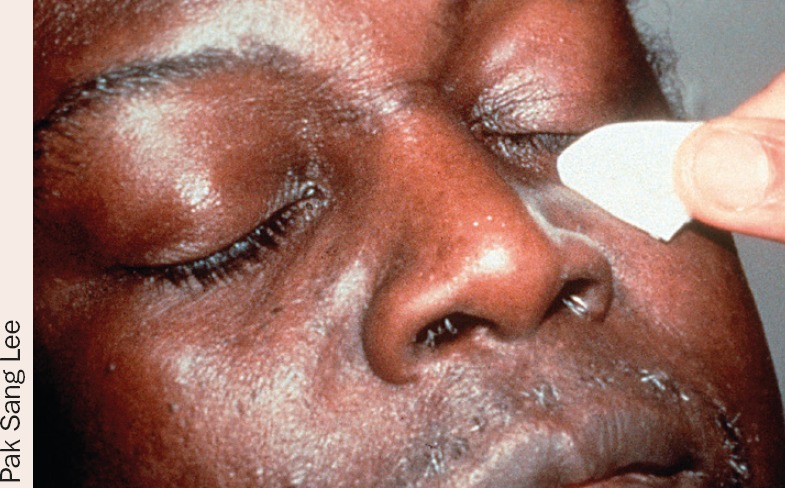


- Ask the patient to close both eyes.
- Take a folded gauze swab.
- Moisten the swab with the prepared solution.
- With the swab, gently clean along the eyelashes in one movement, from inner to outer canthus (Figure 1).
- Discard the swab after use.

**Figure 2:**
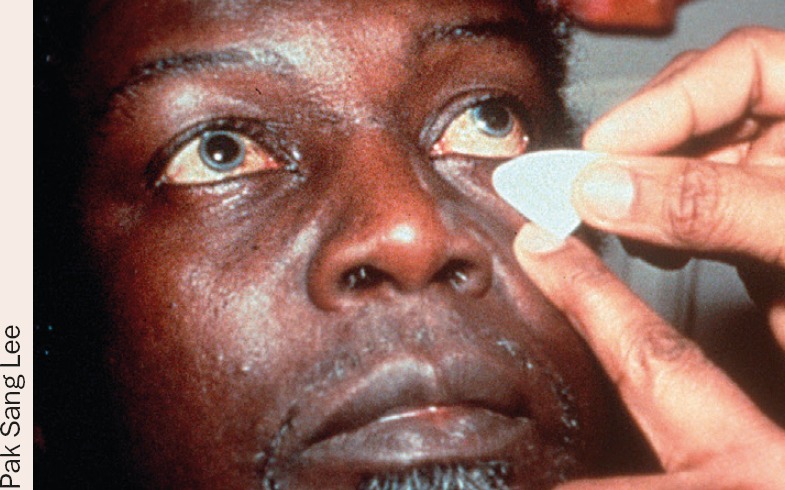


**Figure 3:**
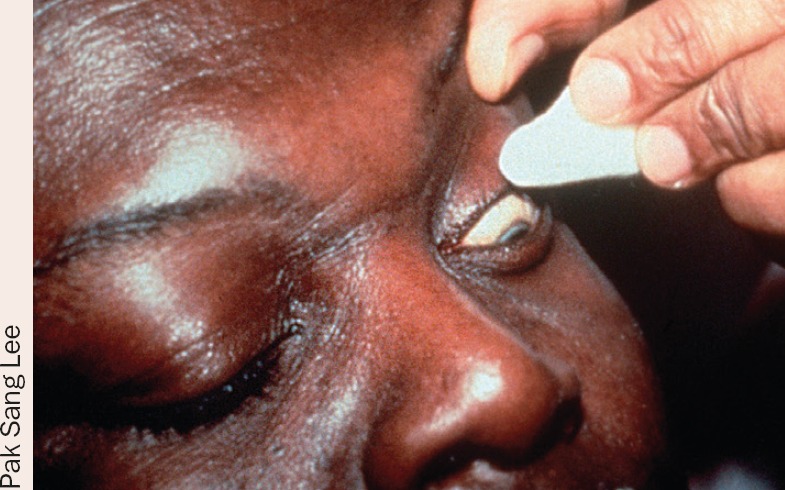


#### 2 The lower eyelid

- Ask the patient to look up.
- With one hand, take a new swab and moisten it in the solution.
- With the index finger of the other hand, gently hold down the lower eyelid.
- With the swab, gently clean along the lower eyelid margin in one movement from inner to outer canthus (Figure 2).
- Discard the swab after use.

#### 3 The upper eyelid

Note: Extra care is needed when cleaning the upper eyelid margin. Try to keep the cornea in view throughout and avoid touching it with the swab.

- Ask the patient to look down.
- With one hand, take a new swab and moisten it in the solution.
- With the thumb or a finger of the other hand, gently ease the upper eyelid up against the orbital rim (just below the eyebrow), taking care not to put any pressure on the eyeball.
- With the swab, gently clean along the upper eyelid margin in one movement from inner to outer canthus (Figure 3).
- Discard the swab after use.

Note: always use a new swab each time

- If the eyelids are very sticky or encrusted, it will be necessary to repeat any part of the above procedure (using a clean swab every time) until all debris or discharge is removed.

Finally, discard the unused remainder of the solution.

## 2 Applying a postoperative dressing

### 

#### You will need

- An eye pad
- An eye shield
- Scissors
- Adhesive tape

#### Preparation

Remind the patient not to open the affected eye under the pad. If the eyelids do not close naturally over the cornea it will be necessary, before padding, to tape the eyelids closed.

#### Method

**Figure 4:**
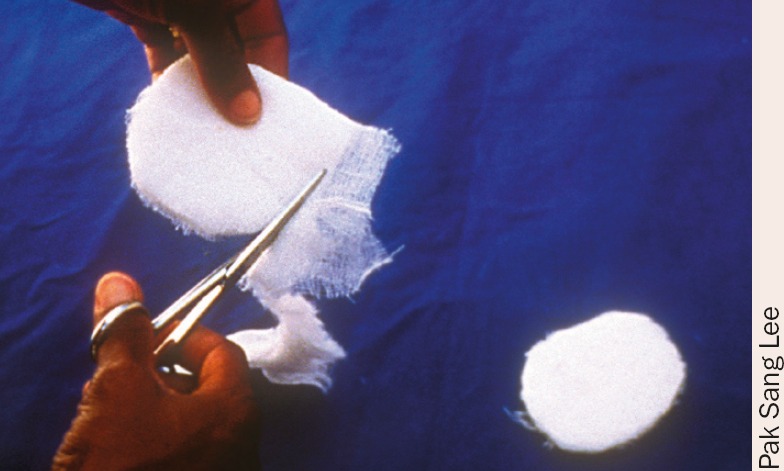


**Figure 5:**
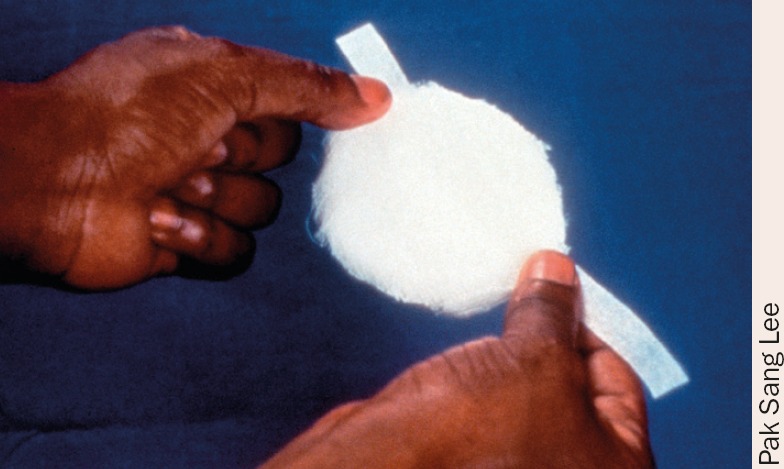


- Use a commercially available eye pad or make your own: place cotton wool between two pieces of gauze and cut into an oval shape approximately 5 centimetres wide and 6 centimetres long (Figure 4).
- Apply a piece of adhesive tape, about 15 cm long, to the eye pad (Figure 5).
- Ask the patient to close both eyes.
- Position the eye pad diagonally over the closed lids of the affected eye and tape firmly, but gently, to the forehead and cheek.
- Apply a second and third piece of tape to ensure the pad lies flat.

**Figure 6:**
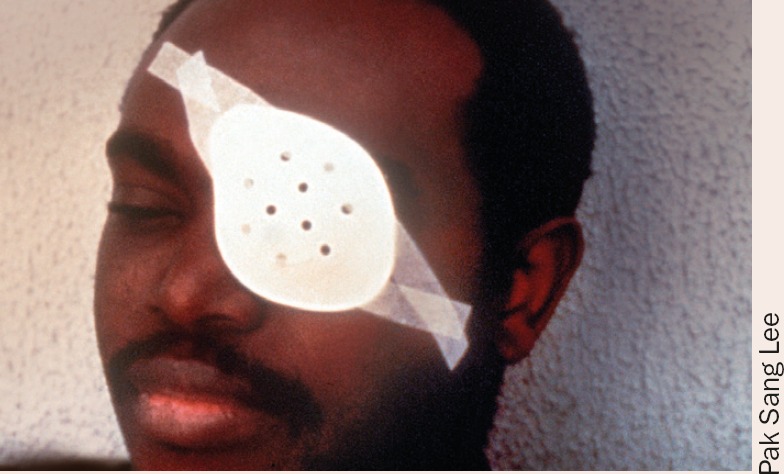


**Figure 7:**
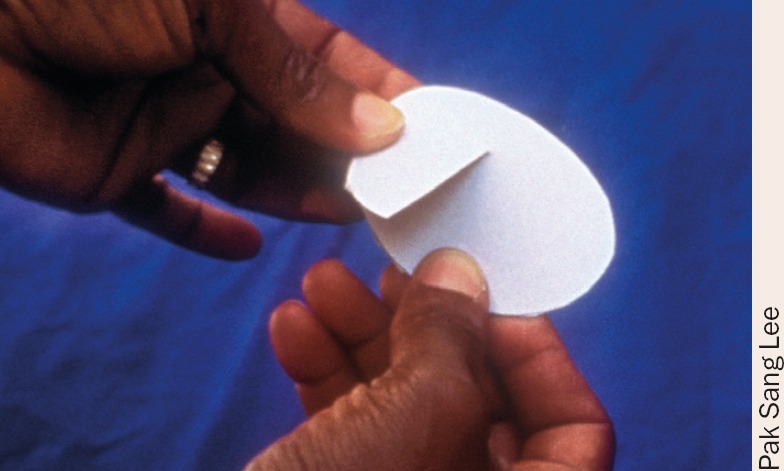


Extra protection can be given by taping a shield over the pad in the same way. The shield in Figure 6 is produced commercially and is called a Cartella shield. You can also make your own. Use a round object to draw a circle approximately 8 cm in diameter on thin cardboard ora used X-ray film and cut around it. Make a single cut into the centre (just half the diameter). Turn into a cone (Figure 7) and secure the shape with adhesive tape.

*Before discharge, show patients how to instil their own eyedrops. This article shows you how:* **www.cehjournal.org/article/instilling-your-own-eye-drops/**

